# Variants in the interferon regulatory factor 5 gene confer genetic risk for systemic lupus erythematosus in a Han Chinese population

**DOI:** 10.1080/07853890.2026.2622737

**Published:** 2026-01-30

**Authors:** Wenqi Xu, Jiayao Liu, Peilin Yu, Xiaofei Shi, Rongzeng Liu

**Affiliations:** a Department of Immunology, College of Basic Medicine and Forensic Medicine, Henan University of Science and Technology, Luoyang, China; b Department of Rheumatology and Immunology, The First Affiliated Hospital, and College of Clinical Medicine of Henan University of Science and Technology, Luoyang, China

**Keywords:** SLE, *IRF5*, single nucleotide variants, haplotype, interferon signaling pathway

## Abstract

**Background:**

Interferon regulatory factor 5 (*IRF5*), integral to interferon signaling pathways, has been identified as a susceptibility locus for systemic lupus erythematosus (SLE). Nevertheless, the relationship between *IRF5* variants and SLE risk within the Han Chinese demographic remains inadequately characterized.

**Materials and methods:**

Genotyping of two functional single nucleotide variants (SNVs) in *IRF5* was conducted in 167 individuals with SLE and 246 healthy controls utilizing sequence-specific primer polymerase chain reaction (PCR-SSP). Chi-square and Fisher’s exact tests were employed to assess associations.

**Results:**

The rs10954213 variant demonstrated a significant association with SLE susceptibility under the recessive model (GG vs. AG+AA, OR = 2.20, 95% CI: 1.30–3.75, *p* = 0.003, adjusted *p* [*p_c_*] = 0.030) and homozygous model (GG vs. AA, OR = 2.43, 95% CI: 1.36–4.42, *p* = 0.003, *p_c_* = 0.032). Similarly, the rs2004640 variant was associated with an increased risk of SLE across allelic (T vs. G, OR = 1.66, 95% CI: 1.22–2.26, *p* = 0.001, *p_c_* = 0.011), dominant (TG+TT vs. GG, OR = 1.77, 95% CI: 1.19–2.63, *p* = 0.005, *p_c_* = 0.047), and homozygous models (TT vs. GG, OR = 3.72, 95% CI: 1.58–8.78, *p* = 0.002, *p_c_* = 0.016). Haplotype analysis identified protective haplotype HT1 (A/G, OR = 0.54, 95% CI: 0.41–0.73, *p* < 0.001) and risk haplotype HT4 (G/T, OR = 2.51, 95% CI: 1.42–4.42, *p* = 0.001).

**Conclusions:**

These findings indicate that *IRF5* gene variants substantially modulate susceptibility to SLE in the Han Chinese population. They hold potential as biomarkers for evaluating SLE risk and offer valuable perspectives into disease pathogenesis.

## 1. Introduction

SLE is a chronic autoimmune disorder marked by immune system dysregulation, the production of autoantibodies, and damage to multiple organ systems, exhibiting considerable clinical heterogeneity [1,2]. The pathogenesis of SLE is attributed to a complex interplay among genetic predisposition, environmental influences, and epigenetic modifications, culminating in a disruption of immune tolerance imbalance and a systemic inflammatory response [3]. Epidemiological evidence indicates a pronounced familial aggregation of SLE, with concordance rates in monozygotic twins ranging from 24% to 56%, which is substantially higher than the 2%–4% observed in dizygotic twins, thereby underscoring the pivotal role of genetic factors in disease development [4–6]. Furthermore, SLE exhibits a marked female predominance, with a female-to-male ratio of approximately 9:1, predominantly affecting women between the ages of 15 and 45, corresponding to their reproductive years [7,8].

Interferon regulatory factors (IRFs) serve as key modulators of type I interferon-stimulated genes and are fundamentally involved in innate immune responses, inflammatory processes, cellular proliferation, and antiviral defense mechanisms [9,10]. Among these factors, *IRF5* has been identified through genome-wide association studies (GWAS) as a significant genetic susceptibility locus for SLE [11–13]. This association is further emphasized by evidence indicating that approximately half of SLE patients display dysregulated gene expression within the interferon signaling cascade, wherein *IRF5* functions as a pivotal pathogenic mediator [14]. The contribution of *IRF5* to SLE pathogenesis is complex and multifactorial. Notably, genetic variants in *IRF5* result in its aberrant overexpression by altering transcriptional regulation or mRNA splicing patterns [12]. Functionally, this upregulation of *IRF5* exacerbates systemic inflammation through the increased production of proinflammatory cytokines, including IL-6 and TNF-α [15]. Additionally, *IRF5* risk alleles facilitate the hyperactivation of myeloid cell populations, such as plasmacytoid dendritic cells (pDCs) and neutrophils, thereby triggering early autoimmune responses that promote B cell differentiation into plasma cells capable of producing autoantibodies [16]. Moreover, *IRF5* directly influences autoantibody production by modulating Toll-like receptor (TLR) signaling pathways within B cells [17–19].

Investigations conducted across diverse geographic populations have consistently demonstrated allelic associations between *IRF5* and SLE, collectively establishing *IRF5* as a significant susceptibility locus for the disease [12,13,20,21]. Notably, the rs2004640 variant, located within the first intron of *IRF5*, generates an alternative splice site, resulting in differential isoform expression and enhanced IFN signaling [12]. Additionally, the rs10954213 variant, positioned in the 3′ untranslated region (3′UTR), influences polyadenylation signals and mRNA stability, thereby modulating the levels of IRF5 protein [22,23]. Numerous studies have examined the relationship between *IRF5* variants and SLE across various populations. For instance, Devaraju P et al. [24] reported that the rs2004640 variant was not associated with an increased risk of SLE, whereas Hammad A et al. [25] found that the presence of the rs2004640 T allele was linked to a heightened risk of the disease. Similarly, the association between the rs10954213 variant and SLE has yielded inconsistent results across different populations [26–28].

These discrepant findings have generated ongoing debate concerning the role of *IRF5* variants in SLE susceptibility. It is plausible that the influence of *IRF5* single nucleotide variants (SNVs) on SLE risk varies among ethnic groups, as studies conducted within Chinese cohorts have produced inconclusive outcomes. In this context, the present case-control study aims to investigate the association between *IRF5* rs10954213 and rs2004640 variants and SLE susceptibility in a Northern Chinese population. By integrating clinical and genetic data, this research provides insights into SLE pathogenesis, thus laying the groundwork for developing precision diagnostics and therapies.

## 2. Materials and methods

### 2.1. Study subjects

Participants were enrolled consecutively from December 2023 through June 2024. This investigation included 167 hospitalized patients diagnosed with SLE of Northern Han Chinese descent, recruited from the Department of Rheumatology and Immunology at The First Affiliated Hospital of Henan University of Science and Technology. All patients satisfied a minimum of four classification criteria established by the American College of Rheumatology (ACR) for SLE diagnosis. Additionally, 246 healthy female control individuals were enrolled from the same geographic region, matched to cases by age, sex, and ethnicity. Control participants had no personal history of autoimmune or systemic diseases, including SLE, and none of their first-, second-, or third-degree relatives had been diagnosed with SLE or other autoimmune conditions. All subjects were unrelated, and cases and controls were matched for both age and geographic origin. Peripheral blood samples were collected from all participants. Among the 167 SLE patients, a subgroup of 122 patients with complete clinical serological records was included for the analysis of demographic and clinical characteristics. Comprehensive medical record reviews were conducted for SLE patients to extract disease-related clinical characteristics, all clinical data collection were completed by August 2024. The study protocol received approval from the Ethics Committee of The First Affiliated Hospital of Henan University of Science and Technology (2023-03-K0049) and was performed in strict accordance with the Declaration of Helsinki. Written informed consent was obtained from all participants prior to enrollment.

### 2.2. SNVs selection

In this study, we selected two functionally relevant SNVs within the *IRF5* gene, namely rs10954213 and rs2004640, based on specific criteria. According to data from the NCBI dbSNP database, both variants possess a minor allele frequency (MAF) of at least 5% in East Asian populations, thereby providing sufficient statistical power for analysis within our target cohort. The rs10954213 was chosen due to its well-documented functional influence on mRNA polyadenylation and stability in the 3′UTR, whereas rs2004640 was selected for its recognized role in modulating alternative splicing of *IRF5* transcripts. Prior functional investigations have demonstrated that these variants significantly affect *IRF5* expression levels and type I interferon signaling pathways. Through this methodical selection approach, we prioritized these two SNVs for detailed examination in our Chinese SLE cohort.

### 2.3. DNA extraction and genotyping

Peripheral blood specimens were obtained from all study participants using EDTA-containing anticoagulant tubes and were promptly stored at −80 °C until further processing. Genomic DNA was isolated from 200 μL of whole blood employing the QIAamp DNA Blood Mini Kit (Qiagen, Germany) in accordance with the manufacturer’s instructions. The concentration and purity of the extracted DNA were evaluated using a NanoDrop 2000 spectrophotometer (Thermo Fisher Scientific, USA), with absorbance ratios (A260/A280) ranging from 1.8 to 2.0 deemed suitable for subsequent analyses. All DNA samples were aliquoted and maintained at −80 °C within a temperature-monitored ultra-low temperature freezer to preserve sample integrity prior to genotyping. For quality assurance, samples exhibiting DNA concentrations below 50 ng/μL or evidence of degradation as assessed by 1% agarose gel electrophoresis were excluded from further analysis.

Genotyping of the *IRF5* rs10954213 and rs2004640 was conducted using the polymerase chain reaction with sequence-specific primers (PCR-SSP) technique. The sequence-specific primers employed in this study (Table 1) were synthesized by Sangon Biotech (Shanghai, China). PCR amplification was carried out in a 20 μL reaction volume comprising 50 ng of genomic DNA template, 10 μL of 2× Taq PCR Master Mix (Sangon Biotech, China), 0.8 μL each of forward and reverse primers (10 μM), and nuclease-free water to reach the final volume. The thermal cycling conditions included an initial denaturation step at 95 °C for 5 min, followed by 34 cycles of denaturation at 94 °C for 30 s, annealing at 59 °C for rs10954213 or 64 °C for rs2004640 for 30 s, and extension at 72 °C for 30 s, concluding with a final extension at 72 °C for 5 min. PCR products were resolved by electrophoresis on 2% agarose gels containing 0.5 mg/mL ethidium bromide and visualized under ultraviolet illumination. The presence of specific alleles was determined based on the detection of target amplification bands.

**Table 1.** Primers used in the genotyping by PCR-SSP.

SNP	Allele	Forward primer	Reverse primer	Product size
rs10954213	A	5′-GCTGAGTCTGTTTTTAACATTT-3′	5′-TTCTCTCCTGGGCTGTCT-3′	397 bp
	G	5′-GCTGAGTCTGTTTTTAACATTC-3′		397 bp
rs2004640	G	5′-ACCCTGCTGTAGGCACCCC-3′	5′-CTCGGTAGTTCGAGCGATTG-3′	198 bp
	T	5′-ACCCTGCTGTAGGCACCCA-3′		198 bp

### 2.4. Statistical analyses

The chi-square test (*χ*^2^ test) was employed to compare differences in genotype and allele frequency distributions of each SNV between case and control groups. Initially, the Pearson chi-square test was conducted to verify whether the genotype distributions of each SNV in the control group conformed to Hardy-Weinberg equilibrium (HWE). Descriptive statistics were utilized to summarize the baseline demographic and clinical characteristics of the study participants, while group comparisons of categorical variables were performed using the *χ*^2^ test. *p* values below 0.05 were regarded as statistically significant. The odds ratio (OR) and corresponding 95% confidence interval (CI) were calculated to quantify the strength of the association between each SNV locus within the *IRF5* gene and susceptibility to SLE. Statistical power analysis was carried out using G*Power 3.1 software to confirm that the sample size was adequate for detecting significant genetic effects. Haplotype construction and linkage disequilibrium (LD) analyses, including the computation of D’ and *r*^2^ statistics, were performed using the online platform SNPStats (https://snpstats.net/start.htm). Additionally, the *χ*^2^ test was applied to examine potential associations between individual *IRF5* SNV loci and specific clinical manifestations in SLE patients. Given the multiple comparisons conducted in these analyses, the Benjamini-Hochberg method was applied to control the false discovery rate (FDR). Associations with an FDR-adjusted *q* value less than 0.05 were considered statistically significant. To ensure transparency, unadjusted *p* values are also reported. Statistical analyses were conducted using SPSS version 26.0 (IBM Corp., Chicago, IL, USA). Furthermore, the Genotype-Tissue Expression (GTEx) portal (https://www.gtexportal.org/home/) was utilized to preliminarily evaluate the potential effects of significantly associated *IRF5* SNVs on gene expression levels.

## 3. Results

### 3.1. Characteristics of the study subjects

The study initially screened 413 individuals and subsequently enrolled 167 female patients diagnosed with SLE alongside 246 age-matched healthy female controls, all of Han Chinese descent from northern China. The demographic and clinical characteristics of the 122 SLE patients with comprehensive clinical data are presented in Table 2. Immunological assessment revealed that 42.6% of the SLE patients were positive for anti-double-stranded DNA (anti-dsDNA) antibodies, while 89.3% tested positive for antinuclear antibodies (ANA). Additionally, 38.5% exhibited reduced complement component C3 levels, 60.7% had decreased C4 levels, 44.3% showed elevated erythrocyte sedimentation rate (ESR), and 17.2% demonstrated increased high-sensitivity C-reactive protein (hs-CRP). Comprehensive clinical features are detailed in Table 2 and included manifestations such as rash (56.6%), photosensitivity (23.0%), oral ulcers (9.8%), alopecia (15.6%), leukopenia (27.9%), arthritis (14.8%), nephritis (51.6%), serositis (13.1%), and neurological disorders (4.9%).

**Table 2.** Demographic characteristics of patients with SLE.

Characteristics	SLE, *n* (%)
Female	122 (100.0%)
Age (mean ± SD; years old)	36.5 ± 11.8
Anti-dsDNA Ab (+)	54 (44.3%)
ANA (+)	112 (91.8%)
Low levels of C3	45 (36.9%)
Low levels of C4	76 (62.3%)
hs-CRP (+)	20 (16.4%)
ESR (+)	57 (46.7%)
Rash	69 (56.6%)
Photosensitivity	28 (23.0%)
Oral ulcer	12 (9.8%)
Alopecia	19 (15.6%)
Leukopenia	34 (27.9%)
Arthritis	18 (14.8%)
Nephritis	63 (51.6%)
Serositis	16 (13.1%)
Neurologic disorder	6 (4.9%)

*Note:* Data correspond to the subgroup of 122 patients with complete and verifiable clinical records. Anti-dsDNA: double-stranded DNA antibody; ANA: antinuclear antibodies; C3: complement 3; C4: complement 4; hs-CRP: hypersensitive-c-reactive-protein; ESR: erythrocyte sedimentation rate.

### 3.2. Genotypic distributions and HWE analysis

Table 3 delineates the genotype and allele frequencies of two *IRF5* SNVs, rs10954213 and rs2004640, within both case and control cohorts, accompanied by the results of HWE testing. The genotype distributions for both SNVs among the control group adhered to HWE assumptions (*p* > 0.05 for each SNV), indicating that the control population is genetically representative. The statistical power to detect the observed associations was calculated to be 76% for rs10954213 and 83% for rs2004640 at a significance threshold of 0.05. Importantly, significant differences were identified in both genotype and allele frequencies of rs10954213 when comparing patient and control groups (*p* = 0.010 and *p* = 0.008, respectively). Likewise, rs2004640 exhibited significant disparities in genotype and allele frequencies.

**Table 3.** Distribution of genotype and allele frequency of *IRF5* gene in controls and SLE group.

Genotype/allele	SLE, *n* (%)	Controls, *n* (%)	*p* value	*p*/HWE
rs10954213 (A > G)				
AA	48 (28.7%)	89 (36.2%)	**0.010**	0.093
AG	81 (48.5%)	128 (52.0%)		
GG	38 (22.8%)	29 (11.8%)		
A	177 (53.0%)	306 (62.2%)	**0.008**	
G	157 (47.0%)	186 (37.8%)		
rs2004640 (G > T)				
GG	70 (41.9%)	138 (56.1%)	**0.002**	0.081
TG	80 (47.9%)	99 (40.2%)		
TT	17 (10.2%)	9 (3.7%)		
G	220 (65.9%)	375 (76.2%)	**0.001**	
T	114 (34.1%)	117 (23.8%)		

*Note:* HWE: Hardy-Weinberg equilibrium. *p* values < 0.05 are indicated in bold.

### 3.3. Association analysis between IRF5 variants and the risk of SLE

This investigation utilized five genetic models, heterozygous, homozygous, dominant, recessive, and allelic, to assess the relationship between SNVs in the *IRF5* gene and susceptibility to SLE (Table 4). Notably, the rs10954213 variant exhibited significant associations with disease risk across several genetic models. Under the allelic model, the G allele was identified as a risk factor for SLE (G vs. A, OR = 1.46, 95% CI: 1.10–1.93, *p =* 0.008). Nevertheless, this association did not remain statistically significant following adjustment for multiple comparisons, yielding a corrected *p*-value (*p*_c_) of 0.060. The recessive model indicated a significantly prevalence of the GG genotype among cases compared to AG and AA genotypes combined (GG vs. AG + AA, OR = 2.20, 95% CI: 1.30–3.75, *p =* 0.003, *p_c_* = 0.030). Similarly, the homozygous model demonstrated that individuals homozygous for the G allele had an elevated risk relative to those homozygous for the A allele (GG vs. AA, OR = 2.43, 95% CI: 1.36–4.42, *p =* 0.003, *p_c_* = 0.032). Conversely, no statistically significant associations were observed under the heterozygous (AG vs. AA, OR = 1.17, 95% CI: 0.75–1.84, *p =* 0.484, *p_c_* = 1.000) or dominant (AG + GG vs. AA, OR = 1.41, 95% CI: 0.27–0.77, *p =* 0.115, *p_c_* = 0.575) models.

**Table 4.** Association analysis of *IRF5* SNVs with SLE.

Polymorphisms	Genetic Model	Genotype	OR (95% CI)	*p* value	*p_c_* value
rs10954213 (A > G)	Allele	G vs. A	1.46 (1.10–1.93)	**0.008**	0.084
	Dominant	AG+GG vs. AA	1.41 (0.92–2.15)	0.115	>0.999
	recessive	GG vs. AG+AA	2.20 (1.30–3.75)	**0.003**	**0.030**
	heterozygous	AG vs. AA	1.17 (0.75–1.84)	0.484	>0.999
	homozygous	GG vs. AA	2.43 (1.36–4.42)	**0.003**	**0.032**
rs2004640 (G > T)	Allele	T vs. G	1.66 (1.22–2.26)	**0.001**	**0.011**
	Dominant	TG+TT vs. GG	1.77 (1.19–2.63)	**0.005**	**0.047**
	recessive	TT vs. TG+GG	2.98 (1.30–6.87)	**0.007**	0.074
	heterozygous	TG vs. GG	1.59 (1.06–2.41)	**0.026**	0.263
	homozygous	TT vs. GG	3.72 (1.58–8.78)	**0.002**	**0.016**

*Note: p_c_*, Bonferroni-adjusted *p* value; *p* values < 0.05 are indicated in bold.

Regarding the rs2004640 variant, the minor T allele demonstrated a significant association with an increased risk of SLE across multiple genetic models. These associations remained statistically significant following Bonferroni correction in the allelic, dominant, and homozygous models. Specifically, within the allelic model, the presence of the T allele was linked to a greater risk compared to the G allele (T vs. G, OR = 1.66, 95% CI: 1.22–2.26, *p* = 0.001, *p_c_* = 0.011). Under the dominant model, carriers of at least one T allele exhibited an increased risk relative to individuals homozygous for the G allele (OR = 1.77, 95% CI: 1.19–2.63, *p* = 0.005, *p_c_* = 0.047). The strongest association was observed in the homozygous model, where individuals homozygous for the T allele (TT) had a markedly higher risk compared to GG homozygotes (OR = 3.72, 95% CI: 1.58–8.78, *p* = 0.002, *p_c_* = 0.016). In contrast, associations identified under the recessive and heterozygous models reached nominal significance but did not withstand Bonferroni correction. Specifically, TT homozygotes showed increased risk in the recessive model (OR = 2.98, 95% CI: 1.30–6.87, *p* = 0.007, *p_c_* = 0.074), while TG heterozygotes exhibited elevated risk in the heterozygous model (OR = 1.59, 95% CI: 1.06–2.41, *p* = 0.026, *p_c_* = 0.263).

### 3.4. Assessing disease susceptibility in SLE through haplotype analysis

Although pairwise LD analysis revealed a weak association between rs10954213 and rs2004640 (r^2^ = 0.045, D′ = 0.405), a comprehensive examination of all potential haplotypes was conducted (Table 5). Notably, the frequency of haplotype HT1 (A/G) was significantly greater in the control group compared to the case group (42.7% vs. 28.7%). This haplotype demonstrated a significant inverse association with SLE risk (OR = 0.54, 95% CI: 0.41–0.73, *p* < 0.001), suggesting a protective role against disease onset. Conversely, haplotype HT4 (G/T) was observed at a significantly higher frequency in cases relative to controls (9.9% vs. 4.3%) and was significantly associated with an elevated risk of SLE (OR = 2.51, 95% CI: 1.42–4.42, *p* = 0.001), indicating its function as a risk factor. In contrast, no statistically significant differences were detected in the frequency distributions of haplotypes HT2 (OR = 1.31, 95% CI: 0.94–1.84, *p* = 0.112) and HT3 (OR = 1.17, 95% CI: 0.87–1.56, *p* = 0.304) between cases and controls, implying that these haplotypes do not contribute to SLE susceptibility.

**Table 5.** Haplotype distribution of *IRF5* gene in patients and controls.

Haplotype	rs10954213	rs2004640	SLE, n (%)	Controls, n (%)	*p* value	OR (95% CI)
HT1	A	G	96 (28.7%)	210 (42.7%)	**<0.001**	0.54 (0.41–0.73)
HT2	A	T	81 (24.3%)	96 (19.5%)	0.112	1.31 (0.94–1.84)
HT3	G	G	124 (37.1%)	165 (33.5%)	0.304	1.17 (0.87–1.56)
HT4	G	T	33 (9.9%)	21 (4.3%)	**0.001**	2.51 (1.42–4.42)

*Note: p* values < 0.05 are indicated in bold.

### 3.5. Immunogenetic analysis of the selected SNVs

Immunogenetic analysis identified several associations between the examined SNVs and specific clinical features of SLE, as summarized in Table 6. Out of the total cohort of 167 SLE patients, a subset of 122 individuals possessing comprehensive clinical and serological data was selected for the immunogenetic evaluation. Specifically, for the rs10954213 variant, patients harboring the AA genotype exhibited a higher prevalence of reduced complement C4 levels compared to those with AG and GG genotypes (65.6% vs. 40.0%, unadjusted *p* = 0.013, *q* = 0.195). Regarding the rs2004640 variant, individuals carrying the T allele demonstrated an increased prevalence of anti-dsDNA positivity (51.5%) compared to GG wild-type homozygotes (35.7%) (unadjusted *p* = 0.008, *q* = 0.120). However, these associations did not retain statistical significance following FDR adjustment for multiple testing (*q* > 0.05 for both). No further significant relationships were identified between these SNVs and other clinical parameters after FDR correction. Moreover, the relationship between haplotypes HT1 (A/G) and HT4 (G/T) and SLE clinical characteristics was evaluated. As presented in Table 7, no significant correlations were detected between these haplotypes and any manifestations of SLE.

**Table 6.** Association of *IRF5* with clinical characteristics of patients with SLE.

Characteristics	rs10954213 (A > G)				rs2004640 (G > T)			
	AA	AG, GG	Unadjusted *p* value	*q* value	GG	TG, TT	Unadjusted *p* value	*q* value
	*n* = 32 (26.2%)	*n* = 90 (73.8%)			*n* = 56 (45.9%)	*n* = 66 (54.1%)		
Anti-dsDNA (+)	16 (50.0%)	38 (42.2%)	0.447	0.981	20 (35.7%)	34 (51.5%)	**0.008**	0.120
ANA (+)	32 (100%)	80 (88.9%)	0.062	0.465	50 (89.3%)	62 (93.9%)	0.350	0.953
Decreased C3	11 (34.4%)	24 (26.7%)	0.408	0.981	17 (30.4%)	28 (42.4%)	0.169	0.953
Decreased C4	21 (65.6%)	36 (40.0%)	**0.013**	0.195	35 (62.5%)	41 (62.1%)	0.966	0.966
Increased hs-CRP	5 (15.6%)	15 (16.7%)	0.891	>0.999	8 (14.3%)	12 (18.2%)	0.562	0.953
Increased ESR	10 (31.3%)	32 (35.6%)	0.660	>0.999	25 (44.6%)	32 (48.5%)	0.672	0.953
Rash	19 (59.4%)	50 (55.6%)	0.708	>0.999	34 (60.7%)	35 (53.0%)	0.394	0.953
Photosensitivity	7 (21.9%)	21 (23.3%)	0.866	>0.999	14 (25.0%)	14 (21.2%)	0.620	0.953
Oral ulcer	3 (9.4%)	9 (10.0%)	>0.999	>0.999	6 (10.7%)	6 (9.1%)	0.764	0.953
Alopecia	5 (15.6%)	14 (15.6%)	0.993	>0.999	9 (16.1%)	10 (15.2%)	0.889	0.953
Leukopenia	9 (28.1%)	25 (27.8%)	0.970	>0.999	15 (26.8%)	19 (28.8%)	0.806	0.953
Arthritis	6 (18.8%)	12 (13.3%)	0.458	0.981	9 (16.1%)	9 (13.6%)	0.212	0.953
Nephritis	14 (43.8%)	49 (54.4%)	0.298	0.981	26 (46.4%)	37 (56.1%)	0.289	0.953
Serositis	5 (15.6%)	11 (12.2%)	0.624	>0.999	3 (5.4%)	3 (4.5%)	0.836	0.953
Neurologic disorder	3 (9.4%)	3 (3.3%)	0.184	0.920	3 (5.4%)	5 (7.6%)	0.622	0.953

*Note:* (+): positive; Anti-dsDNA, double-stranded DNA antibody; ANA: antinuclear antibodies; C3: complement 3; C4: complement 4; hs-CRP: hypersensitive-c-reactive-protein; ESR: erythrocyte sedimentation rate. *p* values < 0.05 are indicated in bold.

**Table 7.** Association of *IRF5* haplotypes with clinical characteristics in SLE.

Characteristics	HT1 (A/G)			HT4 (G/T)		
	+/+	+/−, −/−		+/+	+/−, −/−	
	*n* = 80 (65.6%)	*n* = 42 (34.4%)	*p*	*n* = 40 (32.8%)	*n* = 82 (67.2%)	*p*
Anti-dsDNA (+)	34 (42.5%)	20 (47.6%)	0.589	19 (47.5%)	35 (42.7%)	0.615
ANA (+)	75 (93.8%)	37 (88.1%)	0.279	36 (90.0%)	76 (92.7%)	0.727
Decreased C3	28 (35.0%)	17 (40.5%)	0.551	19 (47.5%)	26 (31.7%)	0.090
Decreased C4	48 (60.0%)	28 (66.7%)	0.470	26 (65.0%)	50 (61.0%)	0.667
Increased hs-CRP	12 (15.0%)	8 (19.0%)	0.566	7 (17.5%)	13 (15.9%)	0.818
Increased ESR	37 (46.3%)	20 (47.6%)	0.885	23 (57.5%)	34 (41.5%)	0.096
Rash	50 (62.5%)	19 (45.2%)	0.068	20 (50.0%)	49 (59.8%)	0.307
Photosensitivity	18 (22.5%)	10 (23.8%)	0.870	8 (20.0%)	20 (24.4%)	0.588
Oral ulcer	6 (7.5%)	6 (14.3%)	0.232	3 (7.5%)	9 (11.0%)	0.749
Alopecia	13 (16.3%)	6 (14.3%)	0.776	7 (17.5%)	12 (14.6%)	0.682
Leukopenia	24 (30.0%)	10 (23.8%)	0.469	12 (30.0%)	22 (26.8%)	0.714
Arthritis	14 (17.5%)	4 (9.5%)	0.291	4 (10.0%)	14 (17.1%)	0.417
Nephritis	38 (47.5%)	25 (59.5%)	0.207	25 (62.5%)	38 (46.3%)	0.094
Serositis	11 (13.6%)	5 (11.9%)	0.774	3 (7.5%)	13 (15.9%)	0.260
Neurologic disorder	4 (5.0%)	2 (4.8%)	> 0.999	1 (2.5%)	5 (6.1%)	0.662

*Note:* (+): positive; Anti-dsDNA: double-stranded DNA antibody; ANA: antinuclear antibodies; C3: complement 3; C4: complement 4; hs-CRP: hypersensitive-c-reactive-protein; ESR: erythrocyte sedimentation rate.

### 3.6. Tissue-sharing and tissue-specific eQTL effects on IRF5 expression

**Figure 1.**
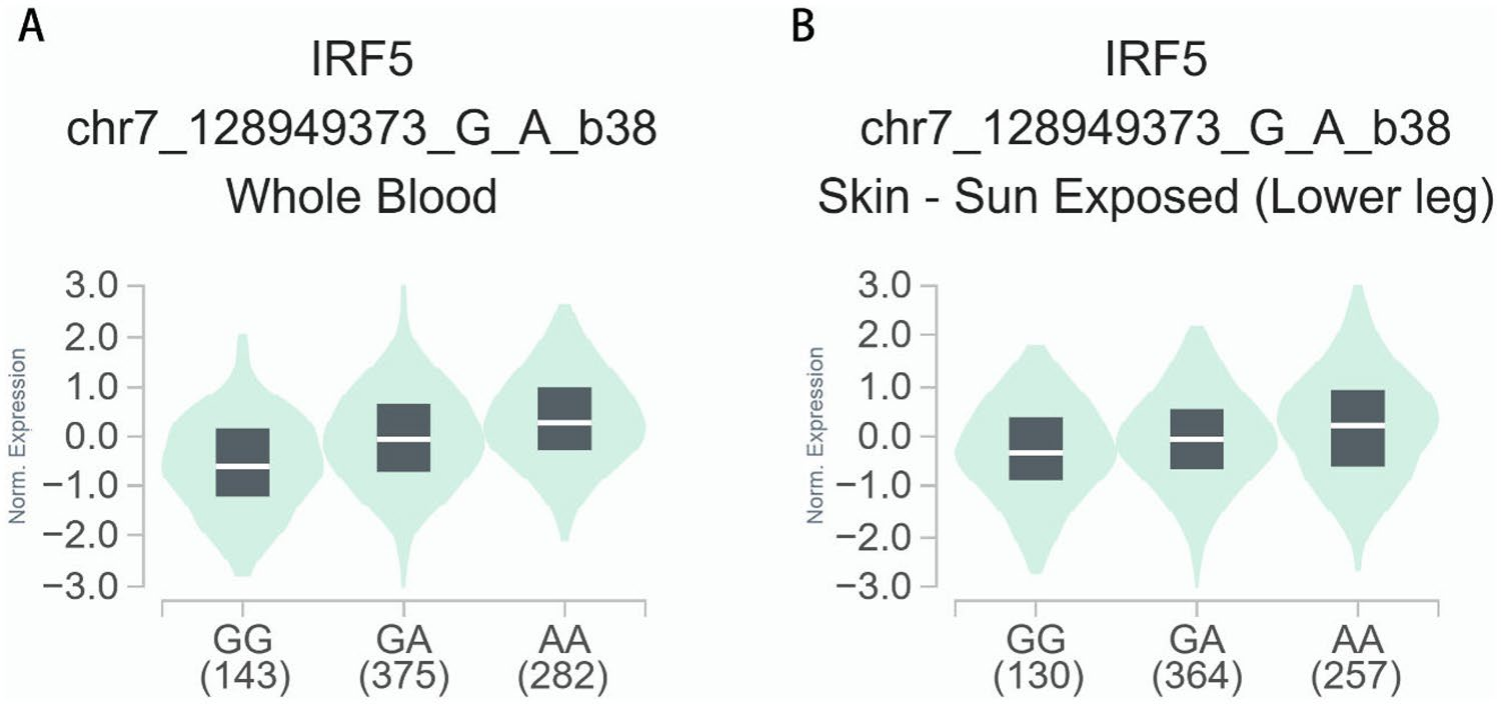
The relationship between the rs10954213 (A > G) variant and expression of *IRF5* examined *via* eQTL analysis of the GTEx database. (A) In whole blood, compared with rs1859168GG/AG genotype, AA genotype was significantly associated with increased *IRF5* gene expression (*p* = 3.18e-83). (B) In skin-sun exposed (Lower leg), compared with rs10954213 GG/AG genotype, AA genotype was significantly associated with increased *IRF5* gene expression (*p* = 1.15e-21).

**Figure 2.**
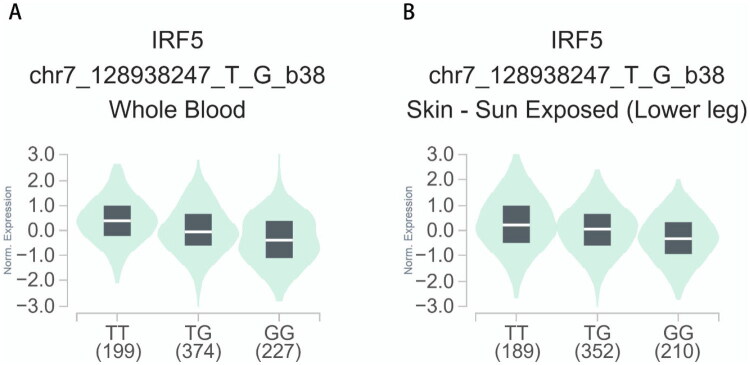
The relationship between the rs2004640 (G > T) variant and expression of *IRF5* examined *via* eQTL analysis of the GTEx database. (A) In whole blood, compared with rs2004640 GG/TG genotype, TT genotype was significantly associated with increased *IRF5* gene expression (*p* = 3.35e-69). (B) In skin-sun exposed (Lower leg), compared with rs2004640 GG/TG genotype, TT genotype was significantly associated with elevated *IRF5* gene expression (*p* = 1.99e-44).

Utilizing expression quantitative trait loci (eQTL) data from the GTEx database, we conducted a comprehensive analysis of the regulatory interactions between two genetic variants, rs10954213 (A > G) and rs2004640 (G > T), and the expression levels of *IRF5*. Tissue selection was determined based on its direct relevance to the pathogenesis of SLE. Whole blood was prioritized to assess gene expression in circulating immune cells, which play a central role in SLE autoimmunity. Additionally, sun-exposed skin from the lower leg was included owing to the clinical significance of photosensitivity, which was observed in 23% of the study cohort. Our findings revealed that the AA genotype of rs10954213 was significantly associated with increased *IRF5* mRNA expression in whole blood (*p* = 3.18e-83, Figure 1A) as well as in sun-exposed skin of the lower leg (*p* = 1.15e-21, Figure 1B). Notably, the TT genotype of rs2004640 exhibited a consistent regulatory effect, as evidenced by substantially increased *IRF5* expression in the corresponding tissues (whole blood: *p* = 3.18e-83, Figure 2A; skin of lower leg skin: *p* = 1.15e-21, Figure 2B). These results indicate that rs10954213 and rs2004640 may serve as regulatory variants influencing *IRF5* expression, thereby potentially contributing to associated biological mechanisms.

## 4. Discussion

SLE is a complex multifactorial autoimmune disorder characterized by the generation of autoantibodies, deposition of immune complexes, and involvement of multiple organ systems. Although the precise etiology of SLE remains elusive, extensive evidence indicates that genetic variants significantly influence individual susceptibility to autoimmune diseases, including SLE [29,30]. IRF5, a pivotal member of the IRF family, is expressed in various immune cells such as B cells, T cells, and dendritic cells. As a critical transcription factor, IRF5 modulates the expression of numerous downstream genes *via* the type I interferon signaling pathway and the Toll-like receptor-myeloid differentiation factor 88 (TLR-MyD88) pathway. This regulatory function positions IRF5 as a central mediator of innate immune responses and implicates it in the pathogenesis of autoimmune conditions, including SLE [31,32].

Notably, *IRF5* rs2004640 (G > T) and rs10954213 (A > G) variants constitute significant genetic risk factors for SLE. The rs2004640 T allele generates a novel splice donor site, thereby promoting the expression of transcripts containing exon 1B and increasing the diversity of *IRF5* isoforms [33]. In parallel, the rs10954213 G allele disrupts a polyadenylation signal within the 3′ untranslated region, resulting in enhanced mRNA stability and elevated IRF5 protein expression [16]. Both variants are associated with augmented activity of the type I interferon pathway, which facilitates autoimmune responses and exhibits consistent correlations with disease susceptibility across diverse populations.

The association between *IRF5* variants and SLE demonstrates considerable variability across different populations and remains a subject of debate. To elucidate the pathogenic role of *IRF5* within the Chinese population, the present study concentrated on two specific SNVs in the *IRF5* gene, rs2004640 (G > T) and rs10954213 (A > G), and systematically examined their association with susceptibility to SLE in this cohort. The findings indicated a significant difference in the genotypic distribution of rs2004640 between the SLE patients and healthy controls, with the TT genotype being significantly correlated with an increased risk of developing SLE, thereby suggesting its role as a risk factor for the disease. Moreover, rs2004640 exhibited notable differences in allele frequencies between cases and controls. The T allele was associated with an increased risk of SLE across multiple genetic models, including dominant and homozygous genetic models.

A considerable body of prior research supports the association of the *IRF5* locus with SLE across various populations, while also emphasizing notable ethnic differences in allele frequencies. For example, the risk-associated T allele of rs2004640, observed at a frequency of 34.1% among our Han Chinese control group, demonstrates a comparatively lower prevalence in Asian populations (32.9%) relative to European populations (56.4%) [34]. Despite this pronounced disparity in baseline allele frequency, the T allele has been consistently implicated in elevated susceptibility to SLE across multiple genetic backgrounds, including European [35], African [36], and Latin American cohorts [37]. The present study extends this well-established association to the Han Chinese population, the largest ethnic group globally. The persistence of this association across populations with varying allele frequencies underscores the variant’s robust pathogenic significance. Moreover, the influence of rs2004640 is not confined to SLE, it has also been associated with rheumatoid arthritis in a Chinese cohort [38] and systemic sclerosis in a meta-analysis [39], indicating its pleiotropic role in autoimmune pathologies. The observed inter-population heterogeneity, exemplified by inconsistent findings in Japanese populations, may be attributable to differences in genetic architecture, environmental exposures, or methodological factors, thereby necessitating further comprehensive investigation.

The rs2004640 variant is situated two base pairs downstream of the exon–intron boundary of exon 1B within the *IRF5* gene, creating a canonical GT donor splice site. Considering the complex transcriptional landscape of *IRF5*, which can be initiated from three alternative promoters (1 A, 1B, or 1 C), this genetic variation may profoundly influence splicing patterns. Empirical evidence indicates that individuals homozygous for the G allele express transcripts exclusively from promoters 1 A and 1 C, but not from 1B, whereas carriers of the T allele co-express transcripts originating from all three promoters (1B, 1 A, and 1 C) [12]. These data imply that specifically the rs2004640 T allele specifically facilitates the constitutive expression of multiple *IRF5* isoforms initiating from exon 1B in plasmacytoid dendritic cells and B cells. This mechanism may modulate IRF5 protein function or stability, potentiate type I interferon pathway responses, and thereby contribute to the development of autoimmunity and the pathogenesis of SLE [12,40,41].

The functional rs1095421 variant, situated within the polyadenylation signal sequence (AATAAA) in the 3′ untranslated region of the *IRF5* gene, constitutes a critical genetic variant influencing its expression. This variant facilitates the production of shorter, more stable mRNA transcripts, thereby markedly elevating overall *IRF5* mRNA levels [22,23]. Its regulatory impact on transcription is particularly pronounced in peripheral blood mononuclear cells [42]. Previous investigations have established significant associations between rs10954213 and susceptibility to autoimmune diseases such as rheumatoid arthritis (RA) [43], multiple sclerosis (MS) [44], and SSc [45]. In the current study, notable differences were detected in the distributions of both allele and genotype frequencies of rs10954213 when comparing SLE patients to control subjects. While the allelic association did not remain statistically significant following adjustment for multiple comparisons, robust associations were consistently observed within particular genetic models. Specifically, the G allele was significantly linked to an increased risk of SLE and demonstrated a strong correlation with heightened susceptibility under recessive and homozygous genetic models. These findings suggest that this variant may contribute to SLE pathogenesis through modulation of *IRF5* expression. Our results align with those reported in European populations [26,46] but contrast with findings from a Japanese cohort as documented by Kawasaki et al. [47]. In recent years, multiple studies have reinforced the allelic association of rs10954213 with SLE, consistently confirming its significant relationship with the disease across several independent cohorts. Collectively, these data indicate that variation at rs10954213 represents a substantial genetic risk factor for SLE, likely exerting its pathogenic influence *via* regulation of *IRF5* transcriptional activity.

Association analysis integrating genotypic and clinical data initially indicated that the rs10954213 variant might be linked to decreased complement C4 levels in patients with SLE. Additionally, the rs2004640 variant exhibited a suggestive correlation with an increased risk of anti-dsDNA antibody positivity. Nevertheless, following FDR correction for multiple comparisons, these associations did not achieve statistical significance (*q* > 0.05), implying that these results should be interpreted with caution as they may represent false-positive findings that warrant validation in larger sample populations. Despite the lack of statistical significance post-correction, these nominal associations pertain to clinical characteristics that have well-established biological connections to *IRF5* function. From a pathophysiological perspective, complement C4, a critical component of the complement cascade, is typically reduced in SLE patients due to increased consumption driven by persistent immune activation. Specifically, immune complexes formed by autoantibodies are cleared *via* complement activation, which involves the consumption of C4, thereby leading to diminished serum C4 concentrations [48]. The T allele of rs2004640 selectively promotes the expression of various *IRF5* isoforms derived from exon 1B. This mechanism may augment type I interferon pathway responses by modifying the activity or stability of the IRF5 protein, thereby contributing to abnormal B-cell activation and disruption of immune tolerance. As a result, this process specifically facilitates the generation of pathogenic autoantibodies, including high-affinity anti-double-stranded DNA antibodies.

The low *r*^2^ value indicates that the SNVs rs10954213 and rs2004640 are likely inherited independently within the studied population. Nonetheless, haplotype analysis uncovers a more intricate genetic framework: despite the weak LD observed between these loci, certain haplotypes, notably HT1 and HT4, exhibit a significant association with disease risk. This suggests that, even in the absence of a strong overall linkage, specific allelic combinations may substantially contribute to disease susceptibility through functional interactions, such as synergistically enhancing *IRF5* transcriptional activity or producing gain-of-function protein isoforms. However, the limited sample size of the current study may constrain the ability to fully characterize the effects of these low-frequency haplotypes, underscoring the need for further validation in larger, independent cohorts.

This investigation reveals that *IRF5* rs2004640 and rs10954213 variants exhibit a significant association with susceptibility to SLE in a Han Chinese cohort. The molecular mechanisms implicated may involve modifications in transcript splicing regulation and mRNA stability, which in turn influence the activity of the type I interferon signaling pathway. Nonetheless, the study is subject to certain limitations. First, our study demonstrated moderate statistical power, approximately 76%, to identify the reported effect sizes associated with rs10954213 at a significance threshold of *α* = 0.05. Although this level of power affords a reasonable degree of confidence in our principal results, it may be inadequate for the reliable detection of smaller genetic effects, especially in subgroup or haplotype analyses. Consequently, the null findings observed in certain analyses, notably those involving haplotypes with lower frequencies, should be interpreted with prudence. Additional limitations of the study include a relatively modest sample size and an incomplete evaluation of all functional variants within the *IRF5* gene. Moreover, the analysis did not account for gene-environment interactions or epigenetic regulatory factors. Future research should aim to replicate these findings in larger, multi-center populations. A critical next step is to experimentally confirm the regulatory mechanisms of rs10954213 and rs2004640, and to functionally characterize the protective haplotype in relevant cell types derived from the Han Chinese population, thereby translating these genetic associations into mechanistic insight. Incorporating such functional assays will be essential to more comprehensively elucidate the biological pathways through which these variants contribute to SLE pathogenesis. Additionally, exploring the relationships between *IRF5* genotypes and clinical phenotypes, therapeutic responses, and long-term prognostic outcomes may yield valuable insights for the precise classification and personalized management of SLE.

## 5. Conclusions

In summary, this study provides evidence that particular variants and haplotypes within the *IRF5* gene are significantly associated with increased susceptibility to SLE in the Han Chinese population. These results underscore the pivotal role of *IRF5* genetic variations in the pathogenesis of SLE and suggest their potential utility as genetic biomarkers for assessing disease risk. Nonetheless, given that this research primarily constitutes a genetic association analysis, further functional investigations are necessary to clarify the specific molecular mechanisms by which these variants modulate SLE progression through downstream signaling pathways. Such insights would offer a theoretical basis for the development of targeted therapeutic strategies aimed at the *IRF5* signaling axis.

## Data Availability

The data presented in this study are available on request from the corresponding author.
